# Case of Concussion of the Brain, with Contusion of the Face, Followed by Paralysis of the Portio Dura Nerve

**Published:** 1838-10-10

**Authors:** 


### PENNSYLVANIA HOSPITAL.

### *Case of Concussion of the Brain, with Contusion of the Face, followed by Paralysis of the Portio Dura Nerve.*

S. B——, a labourer of good habit of body, æt. thirty, was admitted into the hospital on the afternoon of September 7th, 1838, having a short time previously fallen from the fourth story of a store, from which he was attempting to descend by means of a rope extended down the hatchway. When brought in, he was bleeding from the left ear, and had been vomiting; his intelligence was good, he answered several questions well, his pupils were natural and sensible to light. Pulse seventy-six and of moderate force. He complained principally of pain in the left shoulder, and in the left side over the middle ribs, where he had as severe a stitch in breathing, and as much soreness on pressure, as is usually found in cases of fractured rib. Upon examination, some contusion about the left temple was found, severe contusion and pain just in front of the left ear, with fracture of the left clavicle, within the scapular third; there was no fracture of the ribs, neither was there any fracture or depression of the skull. The clavicle being dressed in the usual manner, he was placed in bed, and sinapisms applied to the calves of the legs. In the evening as there was some fever and heat of the head, some cups were applied to the back of the neck, and the effervescing draught given; cold was applied to the head, perfect rest was enjoined, with barley water for diet.

*8th.*—Slight fever; remedies as before.

*9th.*—Considerable fever, severe pain in the head, skin hot, tongue moist and furred, pulse sixty and full, bowels not yet open. Sixteen ounces of blood were taken from the arm, and the following powder ordered:—℞. potass. bit. ℈ij., pulv. jalapæ. gr. x. ft., chart. j. Continue mist. efferves., and ice to head; diet as before.

*10th.*—Much better, less pain in head, skin less hot, pulse sixty and soft, tongue moist, still furred, pupils natural, intelligence good, some noise in the ears, the pain in the side had left him, respiration natural, bowels not being open, a common enema was directed. As he complained of more pain and heat of head in the evening, six cups were applied to the back of the neck. Continue other treatment.

*11th.*—Much better, pulse quicker and soft, tongue good, no heat and but slight pain in head, bowels open. Continue mist. efferves.; diet as before. In evening, owing to a return of pain, five more cups were applied to the neck and temples.

*12th.*—Decidedly better, pulse and skin natural, tongue moist and slightly furred, cerebral symptoms favourable. Blue mass and ipecacuanha followed by castor oil, ordered to open the bowels.

*13th.*—General symptoms good. Observed to-day, for the first time, that the left side of the face is paralysed, the mouth being drawn to the right, and the left eye open. Upon attempting to shut his eyes, he closes the right perfectly, while the left remains about half open; both lower lids being drawn and held down with the same force, he can close only the right, while upon drawing down the upper lids and directing him to raise them, he succeeds equally well in both, showing that the orbicularis muscle, supplied by the portio dura is paralysed, while the levator palpebrarum supplied by a branch of the third pair retains its power of contraction. The motions of the globe of the eye are perfectly natural. In frowning the wrinkle is produced only on the right side, the corrugator muscle of the left being paralysed. The muscles moving the lower jaw seem all to have their proper degree of power, as also those of the tongue, which latter can be protruded in a straight line and moved freely in all directions; states that he cannot close the left side of the mouth so firmly as the right. The sensation on both sides of the face is the same. As he complains of considerable pain just in front of the left ear where he received the contusion, four ounces of blood were ordered to be taken by leeches. Diet, barley water and gruel.

*16th.*—Quite well excepting the paralysis which appears to have been slightly relieved by the leeching. Ordered a small blister to be applied in front of the ear.

*24th.*—The first blister appearing to have a favourable effect, a second was applied on the 26th, which has been decidedly beneficial; he can now with some effort nearly close the eye, the mouth remaining apparently in the same state as before.

*29th.*—Discharged to-day. He can now quite cover the eye, though not without some effort; the mouth also is improved. the deformity having much decreased. The clavicle is firm, no pain or other bad symptoms connected with the brain.

*October 3d.*—Was seen to-day; can close his eyes perfectly, and has almost entirely regained the motion of the left side of his mouth.

This case was thought interesting, from its adding one more to the large number already on record, illustrative of the anatomy of the nerves of the face.

The paralysis was supposed, from the contusion and consequent pain over the track of the nerve, to arise either from inflammation and hardening of the neighbouring tissues, thus causing pressure upon it, or from the inflammation of the nerve itself, and was treated accordingly.

---

The List of Accidents, admitted into the Pennsylvania hospital, during the past fortnight, is to-day omitted. It is intended, hereafter, to publish, in each number, a list of the *discharges* from the surgical wards of the hospital, during the same periods of a fortnight. Our readers will have thus presented to them, not only the character of the numerous interesting accidents and other surgical cases, admitted into the Pennsylvania hospital, with the immediate treatment adopted, but will be, at the same time, in possession of a general outline of the therapeutics of each case, together with its duration and result. The more important cases will, as heretofore, be reported at length.

